# Economic and Productive Comparison of Rutin and Rutin-Loaded Chitosan Alginate Nanoparticles Against Lead-Induced Oxidative Stress in Cobb and Arbor Broiler Breeds

**DOI:** 10.1007/s12011-023-04019-x

**Published:** 2023-12-28

**Authors:** Noha M. Wahed, Mohamed Abomosallam, Basma M Hendam, Zeinab Shouman, Nada MA Hashem, Shimaa A. Sakr

**Affiliations:** 1 Department of Animal Wealth Development, Faculty of Veterinary Medicine, Mansoura University, Mansoura, 35516 Egypt; 2 Department of Forensic Medicine and Toxicology, Faculty of Veterinary Medicine, Mansoura University, Mansoura, 35516 Egypt; 3 Department of Cytology and Histology, Faculty of Veterinary Medicine, Mansoura University, Mansoura, 35516 Egypt; 4 Department of Physiology, Faculty of Veterinary Medicine, Mansoura University, Mansoura, 35516 Egypt

**Keywords:** Rutin, Nanoparticles, Lead, Chitosan, Alginate, Arbor acres breed

## Abstract

Rutin, a natural bioflavonoid compound, is one of the best-known antioxidants. This study aimed to investigate the protective effect of rutin-loaded chitosan alginate nanoparticles (RCA NPs) against lead (Pb)-induced oxidative stress in two different broiler breeds. A total number of 240 chicks from Cobb (CB) and Arbor Acres (AR) breeds were randomly allocated into 4 groups/breed. The 1^st^ group received standard basal diet (SD) and drinking water (DW) while the 2^nd^ group received SD and Pb-incorporated DW (350 mg/L). The 3^rd^ group treated with both rutin-supplemented SD (50 mg/kg feed), and DW contain Pb (350 mg/L). Finally, the 4^th^ group administered RCA NPs-supplemented SD (50 mg/kg feed) and Pb-incorporated DW (350 mg/L). On the 40^th^ day of experiment, broilers weighed, and blood samples collected for biochemical and hematological analysis then slaughtered. Economic efficiency, growth performance, and oxidative stress biomarkers were evaluated. Gene expression level of growth-associated genes as insulin-like growth factor-I (IGF-1) and histopathological changes were assessed in liver and intestinal tissue of both breeds. Our results revealed that Pb-treated birds exhibited the lowest average body weight gain (BWG) and economic efficiency measures in both breeds while RCA NPs-treated groups revealed enhanced growth and economic performance. Furthermore, diet supplementation with RCA NPs considerably enhanced the antioxidant enzymes activity and expression of growth-associated genes than groups treated with rutin alone specifically in AR breed. In conclusion, RCA NPs supplementation could be a promising nanoformulation in poultry production through enhancing the antioxidant capacity and bioavailability of rutin.

**Graphical Abstract:**

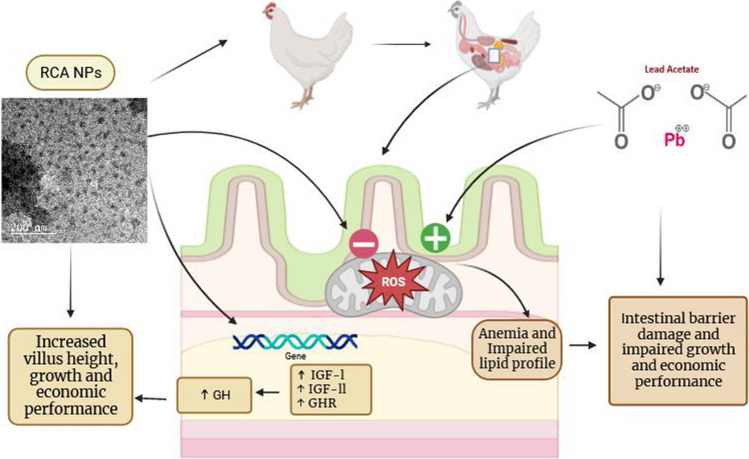

## Introduction

Broilers contribute significantly to human protein needs, because of their shorter life cycle, less capital and land requirements [1]. A major objective of the poultry industry is to continually improve the genetic potential of broiler strains to meet the high-quality with low-cost protein needs of the global population [2]. While producers are seeking a healthy bird with high sale in the shortest possible timeframe, consumers desire tenderness and carcass quality [3]. However, exposure of birds to oxidative stress is a leading non-microbial factor that diminishes meat quality by generating excessive free radicals which lead to cell membrane and mitochondrial damage through lipid peroxidation [4]. Moreover, these stressors are known to depress metabolic rates, retard growth, and increase susceptibility to diseases and reproductive dysfunction in birds [5]. Heavy metals, as major environmental stressors, are still a problem for the poultry industry [6]. Pb is a highly toxic heavy metal that can induce various physiological and biochemical dysfunctions in humans, animals, and birds [7, 8]. Birds can get intoxicated by drinking water contaminated with Pb from old pipes or equipment contaminated with building materials [9]. According to [10], Pb could induce oxidative stress in birds that become weak, ataxic, lose their appetite, lose weight, and get anemic [11]. A chronic Pb exposure causes motor nerve degeneration and peripheral nerve loss, muscle atrophy and even trace levels retard growth and make feed inefficient, resulting in significant economic losses in poultry farms [12]. Nowadays, feed additives are used to exclude the heavy metals-induced oxidative damage, but these additives should not have any negative effects on the performance of birds or on cost-benefit analysis [13].

In recent years, phytogenic feed additives as flavonoids which are natural plant-based bioactive molecules have gained a great deal of attention due to their ability to control diseases beside their economic accessibility and negligible harmful consequences [14], also have potent anti-oxidant properties and performance-boosting effects [15]. Rutin is a flavonoid found in many plants and has numerous benefits, including anti-oxidant, anti-inflammatory, antimicrobial, and antitumor properties [16]. Rutin also commonly used in birds to boost their growth and improve their meat quality because of their widespread availability and safety [17, 18]. Unfortunately, rutin has a major disadvantage due to its poor bioavailability and stability, which may inhibit its biological activity, so it needs to be administered at higher doses to exert its antioxidant effect [19]. Emerging the field of nanotechnology offered new incites in flavonoids delivery systems through modulation of their pharmacokinetic properties which could markedly enhance their clinical potential as potent anti-oxidant agents [20]. Chitosan alginate nanoparticles considered a new natural polymeric vehicle that could improve bioavailability, bioadhesion, targetability, and sustained release of drugs [21]. Furthermore, chitosan nanoparticles could improve young broiler chicken growth performance, production characteristics and intestinal morphology evidenced by hypertrophied villi and epithelial cells [22]. Moreover, chitosan nanoparticles can serve as a chelating agent with a strong affinity for metal ions as Pb [23]. The coupling of alginate, a natural polyanionic polysaccharide, to chitosan nanoparticles can enhance drug uptake via raising the surface charge and could serve as a promising carrier for flavonoids delivery to target tissues [24].

Therefore, this study was designed to evaluate the protective effect of rutin RCA NPs against Pb-induced oxidative stress in terms of economics, growth performance, molecular, biochemical, and histopathological changes, also to compare CB and AR broiler breeds’ responses to Pb-induced oxidative damage.

## Materials and Methods

### Rutin-Loaded Chitosan Alginate Nanoparticle Synthesis

Chitosan alginate nanoparticles were prepared through electrostatic gelation method with a slight modification according to [25]. In brief, chitosan (SRL Ltd., Maharashtra, India) 1% (w/v) was dissolved in diluted acetic acid 1% (v/v) then stirred with rutin (Acros Organics, New Jersey, USA) ethanolic solution (10 mg/ml) for 1 h at 500 rpm. Afterwards, Sodium tripolyphosphate (Piochem, Giza, Egypt) 0.67% (w/v) was added to the above mixture and stirring continued for 2 h. Chitosan-loaded rutin nanosuspension was centrifuged at 5000 rpm for 30 min and then stirred with sodium alginate (SRL Ltd., Maharashtra, India) 0.5% (w/v) for 30 minutes at 500 rpm. Crosslinking of chitosan-loaded rutin nanoparticles with alginate was achieved through addition of CaCl_2_ (0.6 M) followed by stirring for 15 min at 250 rpm; then, the resulting mixture was centrifuged at 5000 rpm for 30 min and dried at hot air oven for 12 h at 60 °C for further analysis.

### Characterization of Rutin-Loaded Chitosan Alginate Nanoparticles

RCA NPs morphology was characterized through transmission electron microscope whereas a drop of diluted sample was deposited and fixed on a copper grid before analysis [26]. Dynamic light scattering (DLS) was used to assess droplet size (nm), zeta potential (mV), and polydispersity index (PDI) of RCA NPs using a Zetasizer Nano ZS (ZEN3600, Malvern Ltd., UK), and three independent measurements were taken of RCA NPs at 25 °C.

### Experimental Design, Diets, and Management

Housing, management, and all birds’ related procedures were conducted according to the guidelines of Mansoura University Animal Care and Use Committee (MU-ACUC), No. (VM.R.22.11.28). A total of 240 1-day-old broiler chicks, 120 CB and 120 AR breed, were purchased from a commercial company (Mansoura Poultry Company, Mansoura, Egypt) and randomly divided into 4 groups/breed with 6 replicates/group and 5 chicks/replicate. Chicks were maintained in adequately ventilated, littered room with a density of 10 birds/m2 under uniform management, hygiene, and housing conditions. A 23L: 1D lighting program was provided on arrival then gradually decreased to 16L: 8D by day 24 until slaughter, and temperature was maintained at 33°C in the first 3 days then gradually decreased by 3°C per week until 25°C. Feed and water were provided ad libitum. All birds were vaccinated against Newcastle disease (ND) and infectious bronchitis (IB) at 7^th^ day of age (MEVAC HB1 + H120, MEVAC Co., Egypt) then revaccinated against ND at 15^th^ day of age (MEVAC ND, MEVAC Co., Egypt), and avian influenza at 10^th^ day of age (MEFLUVAC, MEVAC Co., Egypt) and infectious bursal disease (IBD) at 18^th^ day of age (UNIVAX-BD, MSD Co., USA) following the manufactures’ protocols.

Formulation of diets was based on National Research Council nutrient recommendations [27], as the feeding program consists of three phases: starter (0–14 days), grower (15–28 days), and finisher (29–40 days). Initially, birds were given a corn-soybean meal-based starter ration with 2950 Kcal.ME/Kg, 23% C.P, 2.13% C.F., and 3.35% EE from day followed by the grower ration containing 3000 Kcal.ME/Kg, 21.5% C.P., 2.4% C.F., and 4% EE. The final phase provides them with a finisher ration that contains 3050 Kcal.ME/Kg, 21% C.P., 2.44% C.F, and 4.04% EE until rearing is complete.

Chicks were allocated into 4 treatment groups for each breed as follows;

- The 1st group (control); received SD and DW without treatment.
- The 2nd group (Pb) as lead acetate (Tianjinzhiyuan Chemical Reagent Co., Ltd. Tianjin, China); received SD and Pb-incorporated DW (350 mg/L), according to [28].
- The 3^rd^ group (rutin + Pb); received both rutin-supplemented SD (50 mg/kg feed), according to [29, 30] and DW contain Pb (350 mg/L).
- The 4^th^ group (RCA NPs + Pb); received both RCA NPs-supplemented SD (50 mg/kg feed) and Pb-incorporated DW (350 mg/L).

Chickens were inspected daily for any disorders until time of slaughtering (40 day) and feed intake, individual BW, and feed conversion ratio per pen were recorded weekly.

### Economic Efficiency

On day 40 after rearing started, all birds were sold and economic data were collected for analysis [31].

#### Costs of Production

Bird’s production costs were calculated based on three categories including total variable costs (TVC), total fixed costs (TFC), and total costs (TC) [32, 33].

Feed cost/ bird plus cost of supplements (rutin or RCA NPs) consumed/bird per Egyptian pound (LE) based on the market price at time of experiment calculated as feed cost, while TVC/ bird equal feed cost plus chick cost .

TFC as stated by [34, 35] consists of litter, labor, veterinary management (drugs and vaccines), water, electricity, building and equipment rent, transportation, and miscellaneous costs, plus the cost of the birds purchased [36, 37] (all costs based on market price at experiment time) [38].

$$\textrm{TC}=\textrm{TVC}+\textrm{TFC}.$$

#### Total Returns (TR) [39]

$$\textrm{Total}\ \textrm{returns}\ \left(\textrm{TR}\right)/\textrm{bird}=\textrm{litter}\ \textrm{sale}/\textrm{bird}+\textrm{body}\ \textrm{weight}\ \textrm{sale}/\textrm{bird}$$

$$\textrm{Body}\ \textrm{weight}\ \textrm{sale}/\textrm{bird}=\textrm{marketing}\ \textrm{body}\ \textrm{weight}\ \textrm{of}\ \textrm{bird}\times \textrm{price}\ \textrm{of}\ \textrm{Kg}\ \textrm{in}\ \textrm{market}\ \textrm{at}\ \textrm{time}\ \textrm{of}\ \textrm{experiment}.$$

#### Net Profit (NP) [32, 40]

$$\textrm{NP}/\textrm{bird}=\textrm{total}\ \textrm{returns}\ \left(\textrm{TR}\right)/\textrm{bird}-\textrm{total}\ \textrm{costs}\ \left(\textrm{TC}\right)/\textrm{bird}$$

#### Economic Efficiency Measurements

In accordance with [37], efficiency measures were calculated as percentages of total return to total costs (TR/TC), total cost to total return (TC/TR), net profit to total cost (NP/TC), and net profit to total return (NP/TR).

### Growth Performance

The average live body weight (BW) was recorded weekly; also, the average body weight gain (BWG) was measured for each replicate within groups by subtracting BW at end and start of the rearing periods. Furthermore, the average daily weight gain was also calculated (ADG= BWG/number of days) according to [41, 42]. Moreover, relative growth rate (RGR) was evaluated as ascribed by [43] where RGR = ((W2−W1)/ (0.5 (W2+W1)) ×100 as W1 = initial weight and W2 = final weight.

Feed intake was calculated at the end of each week during experiment considering the number of the dead chicks plus the number of days they fed; then, the feed conversion ratio (FRC) was calculated for each replicate within each group for each feeding phase according to the method reported by [44] whereas FCR = (feed intake / BWG)**.**

### Collection of Samples

At 40 D of age, the feed was deprived for 12 h, 3 broilers per replicate were randomly selected and weighed, and blood samples were collected immediately from the wing vein with and without anticoagulant for hematological and biochemical analysis, respectively; then, birds were slaughtered (*n* = 18 per group). Liver and intestinal samples were dissected and rinsed with saline and liver samples were divided into three parts. The first part was homogenized in ice-cold phosphate buffer saline (pH 7.4) then centrifuged (3000 rpm for 30 min at 4 °C) and supernatant was collected then stored at −80 °C for oxidative stress analysis. The second part stored immediately at −80 °C for molecular analysis while the third part was fixed in neutral buffer formalin (10%, pH 7.0) for histopathological examination.

### Hematological and Biochemical Analysis

#### Hematological Examination

Blood samples with anticoagulant were used for the determination of erythrocyte count (RBCs) and hemoglobin (Hb) concentration according to [45] using an automated hematology analyzer (Sysmex KX-21N).

#### Biochemical Analysis

The concentrations of total protein (TP), total triglycerides (TG), total cholesterol (TCh), high-density lipoprotein-cholesterol (HDL-C), low-density lipoprotein-cholesterol (LDL-C) in serum samples were measured by colorimetric methods with commercial diagnostic kits (Biosino Biotechnology and Science Inc., Beijing, China) through biochemical analyzer (Hitachi Modular System, Hitachi Ltd., Tokyo, Japan).

#### Growth Hormone (GH) Measurement

GH was determined in chickens’ serum samples by ELISA technique with commercially available kits (CSB-E09866Ch), (Cusabio Technology LLC, Houston, TX 77054, USA). GH concentrations were measured in serum samples according to the manufacturers’ instructions as pg/ml at 450 nm through a micro-plate ELISA reader (STAT FAX - 2100) with 625 pg/mL sensitivity and a detection limit ranged from 625 to 10000 pg/mL [46].

### Assessment of Oxidative Stress and Antioxidant Parameters

Lipid peroxidation (LPO) was measured in terms of malondialdehyde (MDA) production which was evaluated following the method reported by [47]; the spectrophotometric absorbance was recorded at 535 nm; then, MDA levels were recorded as nmol/g tissue. GSH content was measured through colorimetric spectrophotometric assay reported by [48]; then, supernatant absorbance was recorded at 412 nm and expressed as mg/g tissue. Catalase (CAT) activity was assayed as reported by [49] based on the decomposition rate of H_2_O_2_ that measured at 240 nm and CAT activity was expressed as units/gm tissue.

The activity of superoxide dismutase (SOD) was assayed according to [50] based on reduction of nitro blue tetrazolium (NBT) into blue formazan and absorptance was recorded at 560 nm; SOD activity was recorded as U /g tissue. Glutathione peroxidase (GPx) activity was determined using H_2_O_2_ as substrate following the method reported by [51] since the reaction was monitored spectrophotometrically at 240 nm and GPx activity was expressed as U /g tissue.

### Quantitative RT-PCR Analysis

TRIzol reagent (Thermo Fisher Scientific, USA, (15596018) was used to extract RNA from frozen liver tissues. RNA was first extracted by homogenization in TRIzol reagent. At 260 and 280 nm absorbance, Nano Photometer® spectrophotometer was used to check concentration and purity of RNA. In the next step, we synthesized cDNA from isolated RNA through Quantitect® Reverse Transcription kit (Qiagen, Germany) according to the manufacturer’s instructions. The target genes were then amplified using forward and reverse primers, their sequences and GenBank accession numbers were listed in Table 1 along with glyceraldehyde 3-phosphate dehydrogenase (GAPDH) as a housekeeping gene (internal control) for normalizing expression levels. Quantitative real-time PCR (qRT-PCR) was used to measure the expressions of IGF-I, IGF-II, GHR, and IGFBP genes in liver tissues using a Rotor-Gene Q instrument and QuantiTect® SYBR® Green PCR kit (Qiagen, Germany). The amplification conditions were 95 °C for ten minutes, followed by 40 cycles of 15 s at 95 °C, 30 s at 60 °C, and 30 s at 72 °C. As described by [52], each gene expression pattern was calculated through the comparative 2-ΔΔCt method.

**Table 1:** List of primers used in RT-PCR reactions

Gene	Primer sequence	GenBank accession no.	Product size (bp)	Reference
*IGF-I*	F:5′-GGTGCTGAGCTGGTTGATGC-3′ R:5′CGTACAGAGCGTGCAGATTTAGGT-3′	JN942578	203	Bhanja and *Kuhad,* (2014)
*IGF-II*	F:5′-GGCGGCAGGCACCATCA-3′ R:5′-CCCGGCAGCAAAAAGTTCAAG-3′	NM_001030342.5	215	Bhanja and *Kuhad,* (2014)
*GHR*	R:5′-GAACCAGCAGTGTGCCTTTG-3′ F:5′CCCTCATCCCCTCCTTCCC -3	XM_046934919.1	205	Xu et al., (2019)
*IGFBP*	F:5′-CACAACCACGAGGACTCAAA-3′ R:5′-CATTCACCGACATCTTGCAC-3′	XM_046921155.1	205	Baloza and El-gendy, (2022)
*GAPDH*	F:5′-TCTTCACCACCGCTCAGTTC-3′ R:5′-TATCAGCCTCTCCCACCTCC-3′	NM_204305.1	114	Lu et al., 2014

*IGF-1*, transcriptional analysis of insulin-like growth factor-I; *IGF-II*, insulin-like growth factor-II; *GHR*, growth hormone receptor; *IGFBP*, insulin-like growth factor binding protein; *GAPDH*, glyceraldehyde 3-phosphate dehydrogenase

### Histopathological Examination

Samples from the liver, duodenum, and cecum were taken at the end of the growing period and rinsed with phosphate buffer saline, then fixed overnight in 10% neutral buffered formalin. The samples were then processed to generate blocks that were then sectioned with rotary microtome (4 μm thick sections), taken on glass slides, and stained with hematoxylin and eosin (HE) [53]. Photomicrographs were taken after examination under light microscope, and certain parameters were measured including (duodenal villi length (DVL), duodenal crypt depth (DCD), cecal mucosal fold length (CMFL), cecal mucosal thickness (CMT), and number of intestinal gland (IG)/ field) [54]. Sections of liver were graded based on the extent of the lesion (0-2= unremarkable, 2–4= mild lesion, 4–6 = moderate lesion, 6 or above= severe lesion) [55].

### Statistical Analysis

The data were collected and SPSS statistical software was used for data analysis [56] using a two-way analysis of variance (ANOVA). Breed, group, and breed-group interaction significance were calculated. Differences were considered statistically significant when *P* ≤ 0.01. The Duncan test [57] and the MSTAT program were used to determine letters for interaction and data were presented as mean ± standard error (SE).

### Percentage of Change

To figure out the percentage of change, results of group B (Pb treated group) compared to the starting point group A (the control), while groups C and D (rutin- and RCA NPs-treated groups) results were compared to B group results as original value. Formula by [58, 59] that calculates percentage of change as= ((new value − original value) / original value) ×100 was used.

## Results and Discussion

### Rutin-Loaded Chitosan Alginate Nanoparticle Characterization

**Fig. 1:**
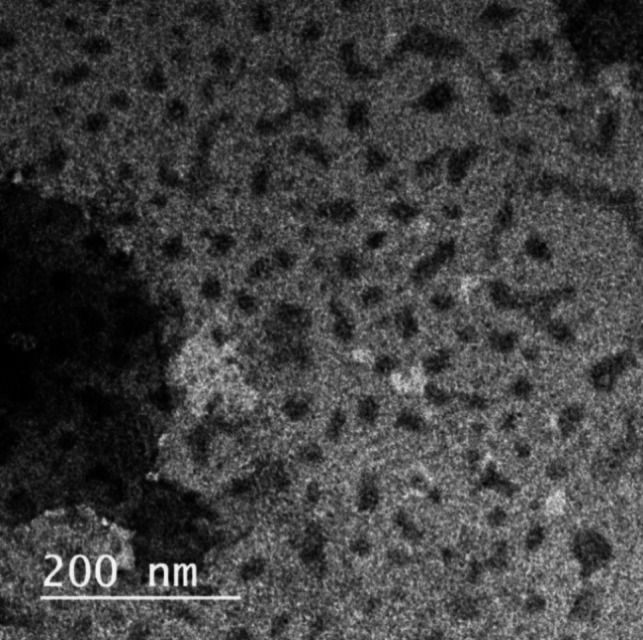
TEM image of RCA NPs

**Fig. 2:**
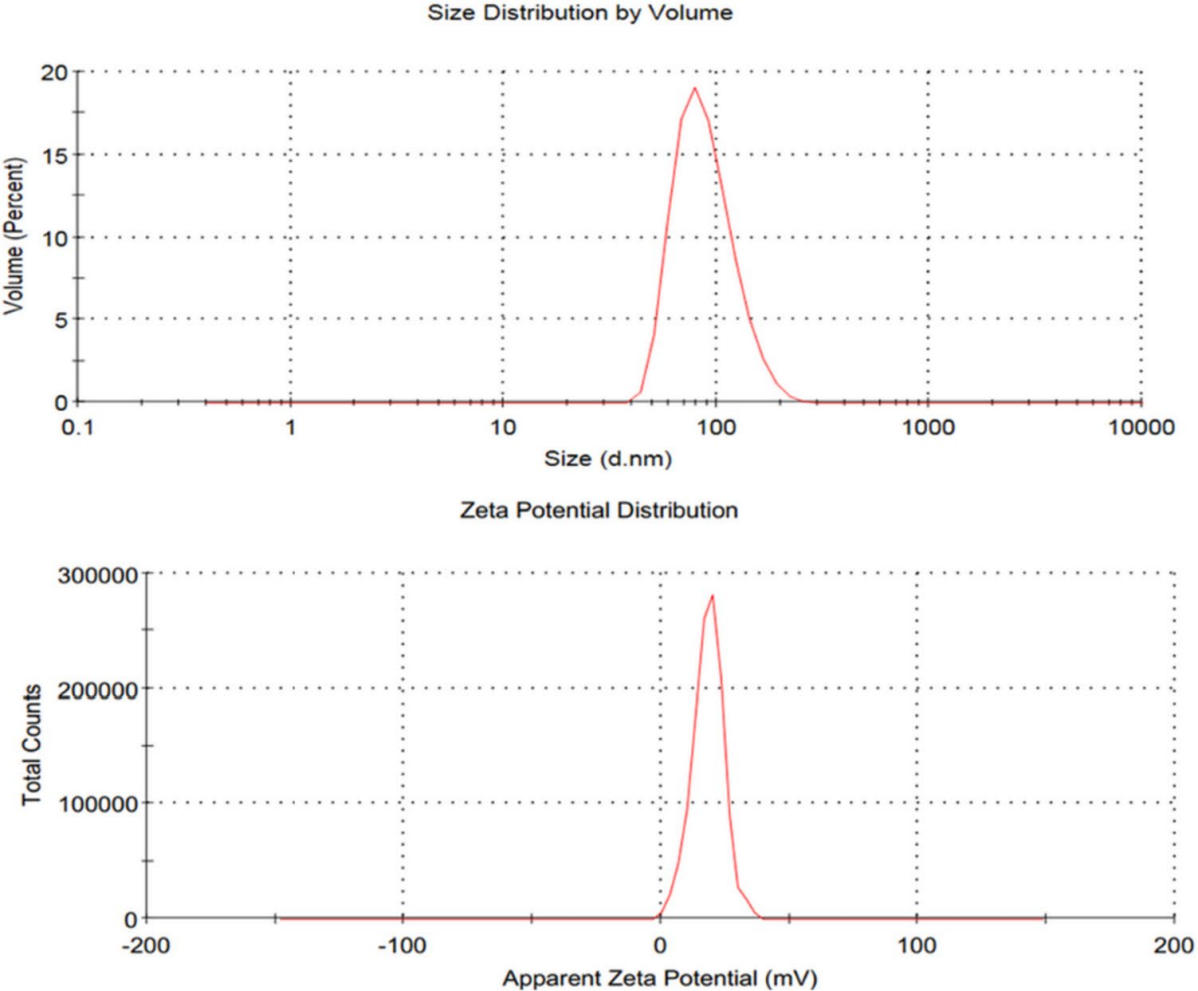
Particle size (nm) and zeta potential (mV) distribution of RCA NPs

TEM micrographs revealed that RCA NPs were spherical, homogenous, and particle sizes ranged from 20 to 50 nm (Fig. 1). The small particle size of RCA NPs could enhance its physical stability and absorption through the gastrointestinal tract [60]. The DLS measurements of RCA NPs in (Fig. 2) revealed average size of RCA NPs was about 90 ± 4.8 nm with PDI of 0.11 ± 0.02 which is relatively larger than that of TEM measurement since TEM analyzes the sample in its dried state with the original size of sample while DLS is a cumulative analysis of scattered light in aqueous medium [61]. The results also confirmed the narrow size distribution, and homogeneity of the prepared nanoparticles as small PDI value (0.11 ± 0.02) indicates homogeneous dispersion of RCA NPs [62, 63]. The zeta potential value was about +18 mV ± 5.2 which is relatively low positive charge confirming that the negatively charged alginate molecules were successfully incorporated on the surface of the positively charged chitosan through electrostatic attraction in a core-shell structure [64].

### Growth Performance

The impact of Pb-induced oxidative stress on the performance of CB and AR breeds varied throughout the six-week experimental period, as shown in Tables 2 and 3. Regarding performance parameters, it was observed that Pb-treated groups exhibited the lowest weekly average BWs, ADG, and FI in both breeds. In comparison to the control groups, final BW values of Pb-treated CB and AR breeds decreased by 36% and 38%, respectively. Additionally, Pb-treated groups showed a substantial decrease in FI accompanied by a significant increase in FCR by 90.98% for CB breed and 94.14% for AR breed when compared to the control groups. Based on these findings, it can be concluded that Pb had a negative impact on broiler growth, including BWG, FI, ADG, and FCR. Similar findings were observed in broiler chicks treated with Pb acetate at dose level 320 mg/kg diet [65], also [66] observed a considerable decrease in BWs of broilers exposed to Pb acetate at a rate of 300 ppm per day compared to the control group. Moreover, parallel findings of lowered growth performance measures were found in broiler chicks treated with Pb acetate at dose level of 400 ppm in drinking water [67]. This decline in performance parameters following Pb exposure may be attributed to alterations in feed consumption and decreased appetites or metabolic disorders, such as inhibition of heme synthesis which leads to cell damage and tissue loss [12], also Pb could induce anxiety, reduction in brain serotonin levels and interruptions of intestinal absorption which all have adverse effect on growth performance [68].

**Table 2:** Effect of Pb, rutin, and RCA NPs on growth performance of Cobb and Arbor acres broiler breeds from 2^nd^ week until 6^th^ week

Item		Cobb breed					Arbor acres breed					*P* value		
		Control	Pb	Rutin + Pb	RCA NPs + Pb	Overall mean	Control	Pb	Rutin + Pb	RCA NPs + Pb	Overall mean	Breeds	Groups	*B*×*G*
Body weight (g)	2nd week	744.2^a^ ± 13.80	692.0 ^a^± 10.76	730.5^a^ ± 7.19	712.3^a^± 2.88	719.8^A^± 3.83	716.0^a^± 6.80	697.0^a^ ± 3.84	711.0^a^ ± 2.64	725.3^a^ ± 5.99	712.3^A^± 3.83	0.93	0.87	0.0001
	3rd week	1392^a^ ± 12.67	1236^c^ ± 7.02	1322^b^ ± 11.23	1364^ab^ ± 10.72	1328^A^± 4.16	1367^ab^ ± 10.91	1246^c^ ± 7.91	1376^ab^ ± 12.69	1395^a^ ± 11.04	1348 ^A^± 4.16	0.0001	0.1	0.0001
	4^th^ week	1934^ab^ ± 11.51	1673^c^ ± 13.73	1927^b^ ± 6.86	1906^b^ ± 5.95	1860^B^± 3.98	1905^b^ ± 12.50	1693^c^ ± 6.36	1997^a^ ± 3.98	1967^ab^ ± 8.62	1890^A^ ± 3.98	0.0001	0.05	0.0001
	5^th^ week	2649^b^ ± 12.54	1782^c^ ± 10.11	2641^b^ ± 8.24	2753^ab^ ± 9.84	2456^A^± 4.06	2673^b^ ± 15.21	1794^c^ ± 11.51	2773^ab^ ± 10.55	2847^a^ ± 13.86	2522^A^± 4.06	0.0001	0.3	0.0001
	6^th^ week	2857^b^ ± 11.43	1820^c^± 13.15	2883^b^ ± 12.79	2955^ab^ ± 12.02	2629^B^ ± 4.73	2897^b^ ± 14.50	1806^c^ ± 12.57	2962^ab^ ± 6.85	3029^a^± 11.99	2673^A^± 4.73	0.0001	0.1	0.0001
Average daily gain (g)	2^nd^–3^rd^ week	92.59^a^ ± 0.96	77.71^b^ ± 0.68	84.50^ab^ ± 1.20	93.10^a^ ± 1.15	87.05^A^± 0.58	92.93^a^ ± 1.12	78.43^ab^ ± 1.37	94.95^a^ ± 1.44	95.67^a^ ± 1.20	90.49^A^ ± 0.58	0.045	0.12	0.18
	3^rd^–4^th^ week	77.43^ab^ ± 0.72	62.43^c^± 1.20	86.50^a^ ± 0.84	77.43^ab^ ± 0.96	75.95^A^± 0.64	76.95^ab^ ± 1.37	63.86^bc^ ± 2.11	88.71^a^ ± 1.31	81.73^a^ ± 1.25	77.82^A^± 0.64	0.001	0.8	0.19
	4^th^–5^th^ week	102.1^b^ ± 3.60	15.57^c^ ± 1.10	102.0^b^ ± 2.29	121.1^ab^ ± 1.20	85.62^A^± 0.95	109.6^ab^ ± 1.20	14.31^c^± 3.39	110.8^ab^ ± 1.37	125.7^a^ ± 2.11	90.10^A^± 0.95	0.0001	0.56	0.0001
	5^th^–6^th^ week	29.67^a^ ± 0.48	5.50^b^ ± 0.92	34.53^a^ ± 1.07	28.73^a^ ± 1.01	24.61^A^ ± 0.59	32.02^a^ ± 1.44	1.76^b^ ± 0.01	27.05^a^ ± 1.12	25.98^a^ ± 2.17	21.7^A^ ± 0.59	0.0001	0.29	0.12

The mean values in the same row with different superscript upper case letters indicate significant differences at (*P* < 0.01). (Small letters for groups and capital litters for breed) *SE*, standard error; *SEM*, standard error of mean

**Table 3:** Effect of Pb, rutin, and RCA NPs on growth performance of Cobb and Arbor acres broiler breeds from 2^nd^ week until 6^th^ week

Item		Cobb breed					Arbor acres breed					*P* value		
		Control	Pb	Rutin + Pb	RCA NPs + Pb	Overall mean	Control	Pb	Rutin + Pb	RCA NPs + Pb	Overall mean	Breeds	Groups	*B*×*G*
Relative growth rate (%)	2^nd^–3^rd^ week	60.65^a^± 1.37	56.43^a^± 2.25	57.77^a^ ± 1.20	62.78^a^± 1.37	60.56^A^± 0.78	62.54^a^± 1.15	56.51^a^± 1.20	64.48^a^± 2.24	63.32^a^± 1.25	61.71^A^± 0.78	0.53	0.27	0.26
	3^rd^–4^th^ week	32.57^ab^ ± 0.92	30.05^ab^ ± 1.15	37.25^a^ ± 1.20	33.15^ab^ ± 1.44	33.26^A^± 0.74	32.93^ab^ ± 1.70	30.42^b^ ± 1.68	36.83^a^ ± 1.20	34.02^ab^ ± 2.21	33.55^A^± 0.74	0.18	0.82	0.0001
	4^th^–5^th^ week	31.20^a^ ± 1.31	6.28^b^ ± 0.58	31.26^a^ ± 1.25	36.35^a^ ± 1.20	25.56^A^± 0.55	33.54^a^ ± 1.25	5.75^b^ ± 0.04	32.53^a^ ± 1.20	36.54^a^ ± 1.31	26.58^A^± 0.55	0.0001	0.0001	0.1
	5^th^–6^th^ week	7.53^a^ ± 0.48	2.14^b^ ± 0.24	8.88^a^ ± 0.50	7.06^a^ ± 0.88	6.39^A^± 0.27	8.02^a^ ± 0.54	0.614^b^ ± 0.01	6.65^a^ ± 0.48	6.18^a^ ± 0.68	5.36^A^± 0.27	0.0001	0.25	0.05
FI	All period	4910^a^ ± 32.31	4850^ab^ ± 37.90	4880^ab^ ± 5.94	4950^a^ ± 11.04	4898^A^ ± 10.47	4840^ab^ ± 21.87	4813^b^± 9.59	4830^ab^ ± 11.99	4.920^a^ ± 12.50	4851^A^± 10.47	0.012	0.0001	0.0001
FCR	All period	2.33^b^ ± 0.06	4.45^a^ ± 0.16	2.27^b^ ± 0.22	2.21^b^ ± 0.23	2.86^A^ ± 0.11	2.22^b^ ± 0.32	4.31^a^ ± 0.31	2.15^b^ ± 0.13	2.14^b^ ± 0.24	2.69^A^± 0.11	0.0001	0.12	0.0001

The mean values in the same row with different superscript upper case letters indicate significant differences at (*P* < 0.01). (Small letters for groups and capital litters for breed) *SE*, standard error; *SEM*, standard error of mean

Conversely, rutin- and RCA NPs-treated CB groups demonstrated a significant increase in final BW by 58% and 62%, respectively, when compared to those exposed to Pb. Similarly, both treatments in AR breed showed an increase in the final body weight value by 64% and 68%, respectively. Furthermore, a significant increase of ADG and FI were observed in rutin- and RCA NPs-treated groups during the fourth and fifth weeks of the experimental period in both breeds. Moreover, a notable improvement of FCR were observed in both breeds with reduction of FCR by 48.99% and 50.34% in rutin- and RCA NPs-treated CB, respectively, and by 50.12% and 50.35% in rutin- and RCA NPs-treated AR, respectively. Improvement of performance parameters in groups treated with rutin and RCA NPs may be attributed to the beneficial effects of flavonoids on the gut morphology and its potent antioxidant properties which promote the bird growth [69, 70]. Furthermore, RCA NPs have a more prominent effect on BWG and FCR than rutin alone since alginate chitosan nanoparticles could be a promising system in rutin delivery as small-sized nanoparticles had extraordinary capabilities to get over many anatomical and physiological barriers and deliver rutin locally to sites of interest, thus enhancing growth performance [71, 72]. Despite the enhanced performance of both breeds following treatment with RCA NPs, AR breed achieved superior outcomes starting from the 4^th^ week of treatment compared to CB breed which agreed with [73] who studied the effects of some phytogenic products on broiler growth performance and concluded that the positive impact of these products is primarily observed during the final stage of growth.

### Economic Efficiency Measures

Tables 4 and 5 present an economic analysis comparing the effects of rutin and RCA NPs on oxidative stress induced by Pb in both CB and AR broiler breeds. No significant differences related to chick, litter, labor, veterinary management, water, electricity, rent, or miscellaneous costs were found based on TFC results for all groups within both breeds. However, when it comes to feed expenses, rutin- and RCA NPs-treated groups in both breeds had the highest expenditure. This can be explained by the additional cost associated with using rutin and RCA NPs, as highlighted by [74] who found that an increase in feed costs and TC were linked to the added expenses of incorporating phytogenic feed additives in broiler diets. Additionally, [75] revealed that feed costs typically make up around 60-70% of production costs in most farms. Therefore, increasing the feeding expenses ultimately led to increased TVC and TC as overall. Furthermore, rising of feed expenses may be attributed to the increased FI of these groups which is consistent with [76] who reported that adding chitosan powder to broiler diets increases feed intake and thus higher feed costs. Hence, it can be concluded that incorporating rutin and RCA NPs into the diet leads to elevated TC/bird.

**Table 4:** Effect of Pb, rutin, and RCA NPs on different economic parameters in Cobb and Arbor acres chicken breeds

Item	Cobb breed					Arbor acres breed					*P* value		
	Control	Pb	Rutin + Pb	RCA NPs + Pb	Overall mean	Control	Pb	Rutin + Pb	RCA NPs + Pb	Overall mean	Breeds	Groups	*B*×*G*
Feed cost/bird	54.01^e^±0.37	53.35^f^±0.37	63.28^c^±0.37	77.38^a^±0.37	62.01^A^± 0.19	53.24^g^ ± 0.37	52.94^h^±0.37	62.73^d^± 0.37	77.05^b^±0.37	61.5^B^± 0.19	0.0001	0.0001	0.0001
Rutin cost	00^b^	00^b^	9.6^a^	9.6^a^	4.8	00^b^	00^b^	9.6^a^	9.6^a^	4.8	0.29	0.001	0.029
Rutin cost/TC	00^e^	00^e^	12.82^b^	10.79^d^	5.9^B^	00^e^	00^e^	12.93^a^	10.83^c^	5.93^A^	0.0001	0.0001	0.0001
Chitosan NPs cost	00^b^	00^b^	00^b^	13.33^a^	3.33^A^	00^b^	00^b^	00^b^	13.33^a^	3.33^A^	-------	0.0001	0.0001
Chitosan NPs/TC	00^c^	00^c^	00^c^	14.98^b^	3.75^B^	00^c^	00^c^	00^c^	15.04^a^	3.76^A^	------	0.0001	0.0001
Chick cost	7	7	7	7	7	7	7	7	7	7	----	----	---
TVC	61.01^e^±0.37	60.35^f^±0.37	69.28^c^±0.37	84.38^a^±0.37	68.76^A^± 0.19	60.24^g^ ± 0.37	59.94^h^±0.37	69.73^d^± 0.37	84.05^b^±0.37	68.5^B^± 0.19	0.0001	0.0001	0.0001
Litter cost	0.83	0.83	0.83	0.83	0.83	0.83	0.83	0.83	0.83	0.83	----	----	----
Labor cost	0.5	0.5	0.5	0.5	0.5	0.5	0.5	0.5	0.5	0.5	----	----	----
Water and electricity cost	0.5	0.5	0.5	0.5	0.5	0.5	0.5	0.5	0.5	0.5	----	----	----
Rent cost	2	2	2	2	2	2	2	2	2	2	----	----	----
VMC	0.45	0.45	0.45	0.45	0.45	0.45	0.45	0.45	0.45	0.45	----	----	----
Miscellaneous costs	0.3	0.3	0.3	0.3	0.3	0.3	0.3	0.3	0.3	0.3	----	----	----
TFC	11.58	11.58	11.58	11.58	11.58	11.58	11.58	11.58	11.58	11.58	----	----	----
TC	54.01^ab^±0.37	53.35^ab^±0.37	53.68^ab^±0.37	54.45^a^±0.37	53.87^A^±0.17	53.24^b^±0.37	52.92^b^±0.37	53.13^b^±0.37	54.12^ab^±0.37	53.35^B^±0.17	0.0001	0.0001	0.0001

The mean values in the same row with different superscript upper case letters indicate significant differences at (*P* < 0.01). (Small letters for groups and capital litters for breed) *SE*, standard error; *SEM*, standard error of mean; *TC*, total costs; *TVC*, total variable costs; *VMC*, veterinary management costs; *TFC*, total fixed costs; *TR*, total returns; *NP*, net profit. Price of chick: 7 LE. Price of kg meat: 35 LE (at time of experiment). L.E: Egyptian Pound

**Table 5:** Effect of Pb, rutin, and RCA NPs on different economic parameters in Cobb and Arbor acres chicken breeds

Item	Cobb breed					Arbor acres breed					*P* value		
	Control	Pb	Rutin + Pb	RCA NPs + Pb	Overall mean	Control	Pb	Rutin + Pb	RCA NPs + Pb	Overall mean	Breeds	Groups	*B*×*G*
Body weight sales	99.99^ab^± 0.47	62.97^b^±0.94	97.77^b^± 1.01	103.41^ab^±0.96	91.03^A^ ± 1.7	101.38^ab^±0.82	63.84^c^±0.27	103.66^ab^±1.01	106.02^a^±0.99	93.73^A^± 1.22	0.11	0.0001	0.12
Litter sales	0.4	0.4	0.4	0.4	0.4	0.4	0.4	0.4	0.4	0.4	---	---	---
TR	100.38^ab^± 0.47	63.37^c^±0.94	98.16^ab^± 1.01	103.81^ab^± 0.96	91.44^A^± 1.16	101.78^ab^ ± 0.82	64.25^c^±0.27	104.06^ab^± 1.01	106.42^a^± 0.99	94.13^A^± 1.65	0.11	0.0001	0.12
NP	34.79^ab^± 0.83	-1.56^f^± 7.79	23.31^cd^±2.24	14.85^e^± 3.47	17.85^B^± 1.22	36.96^a^± 1.51	-0.28^f^± 2.54	29.75^bc^± 2.22	17.79^de^± 2.22	21.06^A^± 1.22	0.06	0.0001	0.32
TC/TR%	65.36^d^±0.40	103.38^a^±1.17	77.65^bc^± 0.99	85.73^b^± 0.98	83^A^± 1.68	63.72^d^±0.70	100.9^a^± 0.34	71.42^cd^± 0.99	83.38^b^±3.4	79.9^A^±1.4	0.11	0.0001	0.19
TR/TC%	153.06^a^±2.76	97.6^d^±4.1	131.13^b^±5.64	116.7^c^±4.39	124.6^B^±8.64	157.02^a^±5.27	99.57^d^±1.82	140.04^b^±5.42	120.1^c^±4.5	129.2^A^±7.6	0.05	0.0001	0.52
NP/TC%	53.06^a^± 2.76	-2.4^d^± 0.3	31.13^b^±5.64	16.70^c^± 4.39	24.6^B^± 1.6	57.02^a^± 5.27	-0.43^d^± 0.3	40.04^b^± 5.42	20.07^c^± 3.3	29.2a ^A^± 1.6	0.05	0.0001	0.09
NP/TR%	34.64^a^± 13.08	-3.38^d^± 4.24	22.35^bc^± 3.89	14.27^c^± 3.86	17^B^± 1.4	36.28^a^± 1.1	-0.9^d^± 0.2	28.58^ab^± 2.7	16.62^c^± 2.7	20.1^A^± 1.4	0.11	0.0001	0.19

The mean values in the same row with different superscript upper case letters indicate significant differences at (*P* < 0.01). (Small letters for groups and capital litters for breed) *SE*, standard error; *SEM*, standard error of mean; *TC*, total costs; *TVC*, total variable costs; *VMC*, veterinary management costs; *TFC*, total fixed costs; *TR*, total returns; *NP*, net profit. Price of chick: 7 LE. Price of kg meat: 35 LE (at time of experiment). L.E: Egyptian Pound

Regarding return measures within groups of both breeds, the BW sale of Pb-treated groups had the lowest value which dropped by about 37% in both breeds compared to the control groups due to the lowest final BW in Pb-exposed groups. This is supported by [77] who displayed that only a small portion of ingested Pb is eliminated through the kidneys while most of it stored in the liver and other vital organs which subsequently leads to oxidative damage, impaired function, retarded growth, and economic losses. On the other hand, the BW sales in rutin-treated groups was compensated by 55.27% and 62.37% in CB and AR breeds, respectively, compared to the Pb-treated group while RCA NPs-treated groups revealed a compensation rate of 64.22% and 66.07% in CB and AR breeds, respectively, compared to the Pb-treated group. This highlighting the significance of incorporating rutin and RCA NPs to compensate losses in the BW sales following exposure to Pb. These findings are consistent with [78] who revealed that adding chitosan at 1 g/kg diet mitigated dexamethasone-induced stress via enhancing growth performance, nutrient digestibility, jejunal morphology, and plasma antioxidant enzymes.

In terms of TR, Pb-treated groups in both breeds experienced a significant reduction in TR by approximately 36% compared to the control groups. However, administering rutin resulted in an increase of TR by 54.89% and 61.96% in CB and AR breeds, respectively. While incorporation of RCA NPs into the broiler diet exhibited a notable increase in TR by 63.82% and 65.63% in CB and AR, respectively, when compared to the Pb-treated groups. These findings suggested that Pb-induced oxidative damage could have a negative impact on TR which counteracted through the dietary supplementation of rutin or RCA NPs. This elevation in TR observed in these treated groups of both breeds could be attributed to the significant rise in their BW sales which coincided with [79] who reported that dietary supplementation of 500 and 1000 mg quercetin/kg diet had improved TR in broilers.

Regarding NP findings, there was a significant decrease of NP in Pb-treated groups compared to the control groups whereas CB and AR breeds experienced reductions in NP by 104.48% and 100.76%, respectively. While addition of rutin and RCA NPs to the broilers’ diet resulted in increased NP levels when compared to Pb-treated groups since rutin-treated groups showed an increase of NP by 1594.23% and 10725% in CB and AR breeds, respectively. However, RCA NPs could enhance NP losses following Pb treatment by 1051.92% and 6453.57% in CB and AR breeds, respectively, which attributed to enhanced FCR, as described in our study, leading to increased BW sales, and ultimately improving NP. These findings supported by [80] who proposed that addition of chitosan in geese diet at 200 mg/kg had beneficial effects on nutrient utilization, digestive enzyme activities, FI, FCR, BW sales, and NP.

Concerning economic efficiency measurements such as TR/TC, TC/TR, NP/TC, and NP/TR ratios, the Pb-treated groups displayed the poorest economic efficiency measures as indicated by higher TC/TR ratio which signified that Pb-induced oxidative stress reduced the economic outcomes through higher costs incurred by lower gains while rutin- and RCA NPs-treated groups improved the economic efficiency measures. Additionally, RCA NPs supplementation cost could be reduced on a large scale through using natural resources of chitosan as shrimp shells that subsequently could improve the overall net profit [81]. Conversely, adding rutin alone in broiler diet may reduce the economic outcomes as higher doses, with subsequently higher costs, are required for rutin to effectively exhibit its antioxidant properties [82]. Thus, using RCA nanoparticles to alleviate oxidative stress on a large scale is a more cost-effective option.

When considering the impact of breed, it was observed that CB breed exhibited higher cost parameters such as feed costs, TVC, and TC along with lower TR compared to AR chickens. Furthermore, AR breed outperformed CB in terms of NP and economic efficiency measures. These findings suggested that AR breed has superior features in tolerating Pb toxicity and displaying favorable economic efficiency measures. Our findings align with the study conducted by [83] which exhibited that the AR breed has better performance under various stress factors compared to the CB breed. Similarly, [84] showed that AR breed demonstrated significantly superior performance compared to the Hubbard breed in all stages of production during the hot season in Saudi Arabia. Moreover, [85] reported that AR breed outperformed CB and Lohmann broiler breeds in a comparative study evaluating the BW sales and NP. These findings can probably be linked to growth-associated genes and genetic makeup variations among different strains [86].

### Hematological and Biochemical Parameters

Hematological parameters as total erythrocytes count (RBCs) and hemoglobin (Hb) concentration were evaluated in both broiler breeds, as shown in Table 6. According to our findings, Pb-treated groups in both breeds showed a significant decrease in RBCs count and Hb concentration (*P*<0.01) compared to the control groups whereas Pb-treated groups dropped RBCs count and Hb concentration by 28.5% and 25.7% in CB chickens, respectively, and by 24.3% and 23% in AR chickens, respectively, when compared to the control groups. These findings coincided with [87] who reported that broilers administered 160 mg/kg Pb acetate showed a significant reduction in RBCs count and Hb concentration which may be because of the fact that Pb can markedly shortened the lifespan of circulating RBCs through interfering with several enzymatic steps in the Hb synthetic pathway and increasing RBCs’ membrane fragility [88]. On the other hand, treatment with rutin and RCA NPs notably raised RBCs and Hb concentration (*P*<0.01) when compared to Pb-treated groups since rutin treatment raised RBCs count and Hb concentration by 14.7% and 13.8%, respectively, in CB breed and by 5.9% and 10% in AR breed compared to the Pb-treated groups. Furthermore, treatment with RCA NPs elevated RBCs count and Hb concentration by 26.2% and 23.7%, respectively, in CB breed and by 23.2% and 18.7% in AR breed as opposed to the Pb-treated groups. These results coincided with [89] who revealed that rutin at dose level of 1 g/kg diet could improve hematological indices in broilers since rutin treatment enhance synthetic pathway of various endogenous proteins including globulins and fibrinogen thus increasing Hb content [90].

**Table 6:** Effect of Pb, rutin, and RCA NPs on hematological and biochemical parameters of Cobb and Arbor acres broiler breeds

Item	Cobb breed					Arbor acres breed					*P* value		
	Control	Pb	Rutin + Pb	RCA NPs + Pb	Overall mean	Control	Pb	Rutin + Pb	RCA NPs + Pb	Overall mean	Breeds	Groups	*B*×*G*
RBCs	2.67^a^ ± 0.16	1.91^c^ ± 0.03	2.19^bc^ ± 0.07	2.41^ab^ ± 0.12	2.29^A^ ± 0.57	2.68^a^ ± 0.11	2.03^c^ ± 0.06	2.15^bc^ ± 0.05	2.50^ab^ ± 0.20	2.34^A^ ± 0.57	0.57	0.001	0.001
Hb	12.48^a^ ± 0.26	9.27^d^ ± 0.50	10.55^bc^ ± 0.16	11.47^b^ ± 0.27	10.94^B^ ± 0.16	12.50^a^ ± 0.21	9.63^cd^ ± 0.47	10.59^bc^ ± 0.17	11.43^b^ ± 0.28	11.3^A^ ± 0.16	0.67	0.0001	0.0001
TP	3.49^a^ ± 0.15	2.19 ^c^ ± 0.15	2.71 ^b^ ± 0.15	3.24 ^a^ ± 0.15	2.908 ^B^ ± 0.07	3.4 ^a^ ± 0.15	2.73^b^ ± 0.15	3.04 ^ab^ ± 0.15	3.23^a^ ± 0.15	3.09^A^ ± 0.07	0.084	0.0001	0.0001
TG	84^d^ ± 3.16	112.67^ab^ ± 3.16	107.67^ab^± 3.16	96.67 ^c^ ± 3.16	100.25^A^ ± 1.58	86^d^ ± 3.16	113.67^a^ ± 3.16	103^bc^ ± 3.16	97.33^c^ ± 3.16	100^A^ ± 1.58	0.912	0.0001	0.0001
TCh	135^c^ ± 3.78	170^a^ ± 3.78	143^bc^ ± 3.78	135^c^ ± 3.78	146.08^B^ ± 1.89	139^c^ ± 3.78	170.33^a^ ± 4.14	155.67^b^ ± 4.14	143.67^bc^± 4.14	152.17^A^± 1.89	0.0001	0.0001	0.0001
HDL-C	38.33^a^ ± 2.48	28.33^c^ ± 2.48	31.33^abc^± 2.48	35^abc^ ± 2.48	33.25^B^ ± 1.24	37 ^ab^ ± 2.48	30^bc^ ± 2.48	35^abc^ ± 2.48	36^abc^ ± 2.48	34.92^A^ ± 1.24	0.356	0.054	0.071
LDL-C	83^d^ ± 2.61	97^ab^ ± 2.61	89.33^bcd^± 2.61	85^cd^ ± 2.61	88.58^B^ ± 1.31	81.67^d^ ± 2.61	100.33^a^ ± 6.89	93^abc^ ± 12.61	84^d^±24.56	89.75^A^ ± 1.31	0.537	0.001	0.001
GH	3.58^a^ ± 0.14	2.63^d^ ± 0.14	2.95 ^cd^ ± 0.14	3.18 ^ab^ ± 0.14	3.087^A^ ± 0.07	3.48 ^ab^ ± 0.14	2.76^cd^ ± 0.14	2.96 ^cd^ ± 0.18	3.13^bc^ ± 0.18	3.082^A^ ± 0.07	0.96	0.001	0.002

The mean values in the same row with different superscript upper case letters indicate significant differences at (*P* < 0.01). (Small letters for groups and capital litters for breed) *SE*, standard error; *SEM*, standard error of mean; *RBCs count*, total erythrocytic count; *Hb*, hemoglobin concentration; *TG*, total triglyceride; *TCh*, total cholesterol; *HDL-C*, high-density lipoprotein; *LDL-C*, low-density lipoprotein; *TP*, total protein; *GH*, growth hormone

Lipid profile including total triglyceride (TG), total cholesterol (TCh), high-density lipoprotein (HDL-C), and low-density lipoprotein (LDL-C) besides total protein (TP) concentration was measured in both broiler breeds (Table 6) and data displayed that Pb-treated groups in both breeds showed a significant increase in TG, TCh, and LDL-C (*P*<0.01), while HDL-C and TP were markedly decreased (*P*<0.01) when compared to the control groups. Pb treatment raised TG, TCh, and LDL-C by 34.1%, 25.9%, and 16.9% in CB broilers, respectively, and by 32.2%, 22.5%, and 22.8% in AR broilers, respectively, while reduced HDL-C and TP by 26.1% and 37.2% in CB broilers, respectively, and by 18.9% and 19.7% in AR broilers, respectively, when compared to the control groups. These findings were in the same line with [91] who reported that exposure to Pb acetate at 100 mg/kg BW in broiler chickens resulted in marked elevation of TCh, TG, and LDL-C Triglycerides through activation of cholesterol-biosynthetic enzymes with suppression of cholesterol-catabolic enzymes resulting in hepatic hypercholesterolemia and hypertriglyceridemia, also low level of HDL-C considered one of the most common lipid abnormalities related to heavy metal exposure [92]. Reduction of TP following Pb exposure was consistent with [93] who observed that Pb administered at 284 mg/kg BW in chicken reduced TP significantly which may be due to liver damage caused by Pb exposure, also degradation of synthesized proteins through Pb acetate action on free amino acids [94].

On the other side, rutin and RCA NPs supplementation significantly reduced TG, TCh, and LDL-C (*P*<0.01), while HDL-C and TP were markedly increased in comparison to Pb-treated groups. Rutin supplementation reduced TG, TCh, and LDL-C by 4.4%, 15.9%, and 7.9% in CB birds, respectively, and by 9.4%, 8.6%, and 7.3% in AR birds, respectively; otherwise, HDL-C and TP were elevated by 10.6% and 23.7% in CB birds, respectively, and by 16.7% and 11.4% in AR birds, respectively, compared to the Pb-treated groups. Correspondingly, RCA NPs supplementation reduced TG, TCh, and LDL-C by 14.2%, 20.6%, and 12.4%, respectively, in CB birds and by 14.4%, 15.7%, and 16.3%, respectively, in AR birds, apart from that HDL-C and TP were elevated by 23.5% and 47.9%, respectively, in CB birds and by 20% and 18.3%, respectively, in AR birds as comparable to the Pb-treated groups. Our data demonstrated that rutin could modulate hypoproteinemia, hypertriglyceridemia, and hypercholesterolemia *via* improving metabolism and structural integrity through its scavenging activity of free radicals besides it may inhibits cholesterol biosynthetic key enzymes as β-Hydroxy β-methylglutaryl-CoA (HMG-CoA) reductase and decrease the amount of circulating free fatty acids (FFA) available for triacylglycerol synthesis [95, 96]. These findings are consistent with [97] who revealed that rutin at dose level 400 mg/kg diet in broilers had a marked hypolipidemic effect.

Plasma GH concentration was also measured in treated chickens of both breeds as shown in (Table 6) and results displayed that Pb-treated groups in both breeds significantly decreased GH concentration when compared to the control groups whereas GH concentration declined by 26.5% in CB broilers and by 20.7% in AR broilers. This decline could be attributed to prolonged exposure to Pb with subsequent inhibition of GH plasma activity [10]. On the other hand, rutin and RCA NPs supplementation enhanced GH concentration in comparison to the Pb-treated groups, since GH concentration increased by 12.2% and 20.9% in CB and by 7.2% and 13.4% in AR, respectively. Based on these findings, the enhancement of GH is strongly connected to the growth performance of birds, as evidenced by significant improvement in BW and FCR, which may be attributed to the stimulation of GH receptors in skeletal muscle, which in turn increases protein synthesis and stimulates skeletal muscle growth and development, leading to better growth performance [98].

### Antioxidant Enzyme Activities and Oxidative Stress Biomarkers

Oxidative damage usually occurs when the antioxidant capability of tissues is overwhelmed by excessive reactive oxygen species (ROS) generation such as superoxide radicals, hydroxyl radicals, and hydrogen peroxides during the metabolic processing of various toxins [99, 100]. The current study investigated the antioxidant defense system in liver tissues of both CB and AR breeds including enzymatic activity of SOD, CAT, and GPx besides non-enzymatic antioxidants as GSH. Additionally, levels of MDA were measured to assess lipid peroxidation, as shown in Table 7. Pb-treated groups of both breeds displayed a significant reduction of GSH, SOD, GPx, and CAT enzymes activity (*P*<0.01) with a marked increase in MDA level (*P*<0.01) when compared to the control groups. When assessing each breed separately, Pb-treated CB chickens exhibited reduction of GSH, SOD, GPx, and CAT levels by 48.5%, 59.7%, 50.6%, and 60.7%, respectively, while MDA level was raised by 184.9% when compared to the control group. Correspondingly, Pb-treated AR chickens displayed decline of GSH, SOD, GPx, and CAT levels by 31.8%, 51.2%, 36.9%, and 44.3%, respectively, while MDA level was elevated by 118.9% when compared to the control group. These results coincided with previous study carried by [101] who revealed that Pb could induce liver oxidative damage at dose level of 350 mg/L in drinking water of broiler chickens since Pb inhibits the sulfur containing antioxidant enzymes via making complexes with the sulfhydryl (–SH) groups with subsequent inhibition of their functional activity in scavenging free radicals and peroxides [102].

**Table 7:** Effect of Pb, rutin, and RCA NPs on oxidative stress in Cobb and Arbor acres broiler breeds

Item	Cobb breed					Arbor acres breed					*P* value		
	Control	Pb	Rutin + Pb	RCA NPs + Pb	Overall mean	Control	Pb	Rutin + Pb	RCA NPs + Pb	Overall mean	Breeds	Groups	*B*×*G*
GSH	22.33^a^ ± 0.71	11.50^d^ ± 0.56	15.0^c^ ± 0.37	19.17^b^ ± 0.84	17.0^B^ ± 0.89	22.0^a^ ± 0.82	15.0^c^ ± 0.37	17.33^b^ ± 0.33	19.0^b^ ± 0.93	18.33^A^ ± 0.62	0.004	0.000	0.006
MDA	19.83^f^ ± 0.6	56.50^a^ ±1.02	44.5^c^ ± 0.76	27.83^e^ ± 0.6	37.17^A^ ± 2.99	22.0^f^ ± 1.39	48.17^b^ ± 1.11	36.5^d^ ± 0.56	26.17^e^ ± 0.79	33.21^B^ ± 2.16	0.000	0.000	0.000
SOD	62.83^a^ ± 0.7	25.33^f^ ± 1.1	34.83^d^ ± 0.79	43.67^c^ ± 0.8	41.67^B^ ± 2.91	60.17^a^ ± 1.01	29.33^e^ ± 0.67	41.33^c^ ± 0.84	49.17^b^ ± 1.01	45.0^A^ ± 2.38	0.000	0.000	0.000
GPx	83.0^a^ ± 1.15	41.0^e^ ± 1.37	51.33^d^ ± 1.28	66.17^c^ ± 1.4	60.38^B^ ± 3.36	80.5^ab^ ± 1.09	50.83^d^ ± 1.14	64.17^c^ ± 0.79	77.17^b^ ± 0.87	68.17^A^ ± 2.49	0.000	0.000	0.000
CAT	43.33^a^ ± 0.99	17.0^e^ ± 0.37	21.83^d^ ± 0.98	31.67^c^ ± 0.84	28.46^B^ ± 2.14	40.67^ab^ ± 0.8	22.67^d^ ± 0.71	31.5^c^ ± 0.43	38.5^b^ ± 0.56	33.33^A^ ± 1.49	0.000	0.000	0.000

The mean values in the same row with different superscript upper case letters indicate significant differences at (*P* < 0.01). (Small letters for groups and capital litters for breed) *SE*, standard error; *SEM*, standard error of mean

In contrast, rutin- and RCA NPs-supplemented groups markedly raised GSH, SOD, GPx, and CAT levels (*P*<0.01) while reduced MDA level (*P*<0.01) compared to the Pb-treated groups in both breeds. Rutin-treated CB chicken revealed enhanced GSH, SOD, GPx, and CAT levels by 30.4%, 37.5%, 25.2%, and 28.4%, respectively, while MDA level reduced by 21.2% when compared with Pb-treated group. Moreover, RCA NPs-treated CB chicken exhibited elevation of GSH, SOD, GPx, and CAT levels by 66.7%, 72.4%, 61.3%, and 86.3%, respectively, while MDA level declined by 50.7% in comparison to the Pb-treated group. Correspondingly, Rutin-treated AR chicken displayed boosted GSH, SOD, GPx, and CAT levels by 15.5%, 40.9%, 26.2%, and 38.9%, respectively, while MDA level decreased by 24.2% when compared with Pb-treated group. Furthermore, RCA NPs-treated AR chicken unveiled marked rise of GSH, SOD, GPx, and CAT levels by 26.7%, 67.6%, 51.8%, and 69.8%, respectively, while MDA level dropped by 45.7% in comparison to the Pb-treated group. These results come in agreement with previous findings of [103] who revealed that rutin at dose level of 500 mg/kg diet displayed potent antioxidant effects since rutin, as a flavonoid, could inhibit xanthine oxidase activity and lipid peroxidation by scavenging free radicals that attributed to its free hydroxyl groups on A and B rings of rutin carbon skeleton [104]. However, rutin major disadvantage related to its poor bioavailability and stability that may hinder its biological activity so necessitate administration of higher doses to exert its antioxidant properties [70]. Chitosan alginate nanoparticles considered a new natural polymeric vehicle that could improve bioavailability, bio-adhesion, target ability, and sustained release of drugs [21]. Furthermore, these nanoparticles could enhance the antioxidant activity of rutin and maintain its pharmaceutical formulations [105]. In respect to difference between breeds, AR breed showed higher resistance to Pb exposure than CB breed which may attributed to the differential feed efficiency and fat deposition under environmental stressors which in turn could be breed-dependent [38].

### Quantitative RT-PCR Analysis

**Fig. 3:**
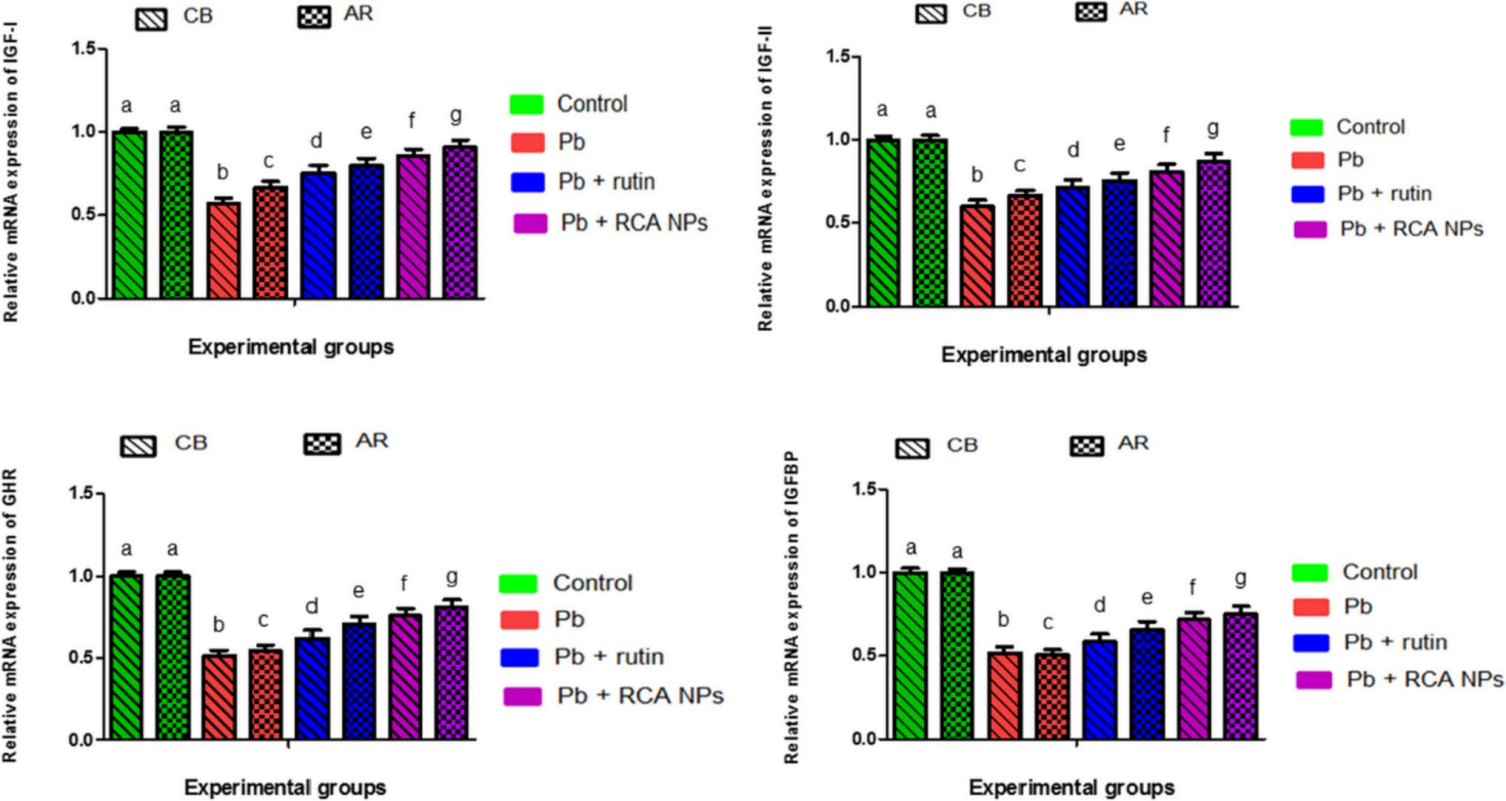
Changes in the mRNA expression folds of hepatic *IGF-I*, *IGF-II*, *GHR*, and *IGFBP* genes in CB and AR broiler breeds fed diets containing Pb, rutin, and RCA NPs. Data analyzed through two-way analysis of variance (ANOVA) and different letters indicate statistical significance at (*P* < 0.01)

Concerning the relative mRNA expression levels of IGF-I, IGF-II, GHR, and IGFBP genes in the broilers’ liver tissues (Fig. 3), it was demonstrated that Pb treatment reduced their expressions significantly (*P* < 0.01) by 42.5%, 39.7%, 48.3%, and 48.2%, respectively, in CB breed, and by 33.3%, 33.7%, 45.3%, and 49.5%, respectively, in AR breed compared to control groups. These results are in agreement with [106] who reported that 12.5 μM of Pb acetate could induce marked downregulation of growth-associated genes as IGF-I since exposure to Pb resulted in excessive generation of reactive oxygen species (ROS) which subsequently lead to DNA destruction and alter the relative mRNA expression patterns of theses growth-associated genes [107]. Hence, growth-associated genes including IGF-I and IGF-II play a significant role in poultry growth, body mass conformation, skeletal development, and fat accumulation which mostly expressed in liver and muscle tissues [108]. Therefore, Pb could induce reduction of growth performance in exposed birds.

Meanwhile, rutin- and RCA NPs-treated groups of both breeds mitigated Pb-induced oxidative damage and notably (*P* < 0.01) enhanced growth-associated genes expression in liver tissue. Rutin upregulated IGF-I, IGF-II, GHR, and IGFBP markedly by 30.96%, 18.24%, 19.92%, and 12.55%, respectively, in CB breed and by 19.64%, 13.88%, 29.25%, and 30.09%, respectively, in AR breed compared to the Pb-treated groups. Correspondingly, RCA NPs were more effective than rutin in reversing oxidative stress induced by Pb as evidenced by significant (*P* < 0.01) upregulation of growth-associated genes. In the RCA NP-treated groups compared to Pb-treated groups, there was an increase in the gene expression levels of IGF-I, IGF-II, GHR, and IGFBP by 49.04%, 33.99%, 46.62%, and 38.42%, respectively, in CB breed and by 35.98%, 31.67%, 48.63%, and 48.52%, respectively, in AR breed. These findings suggested that rutin quenches the toxicity of Pb acetate though improving the antioxidant mechanisms and serving as a scavenger of free radicals by depressing expression of oxidative stress genes [109], thus decreasing DNA and protein damage that is provoked by ROS generation [110]. Our data also revealed the molecular mechanisms following RCA NPs incorporation into the diet with subsequent enhanced growth performance and economic outcomes as the nutritional status regulates the levels of circulating IGF-I and IGF-II and their expression [111]. Furthermore, GHR plays an important role in improving production traits as it affects GH activity mainly through forming GH-GHR complexes which in turn activate IGF-I secretion in the liver [112]. Moreover, IGFBP plays a crucial role in modulating the action of the IGFs by controlling the half-life of the IGFs in circulation so that a balance is struck between good and bad signals that regulate bird growth rate through development of muscle [113]. Regarding breed effect, AR breed is superior than CB breed as evidenced by significant upregulation of growth-related genes which may be related to the genetic makeup differences among breeds [114].

### Histomorphometric Analysis of Intestine and Liver

**Fig. 4:**
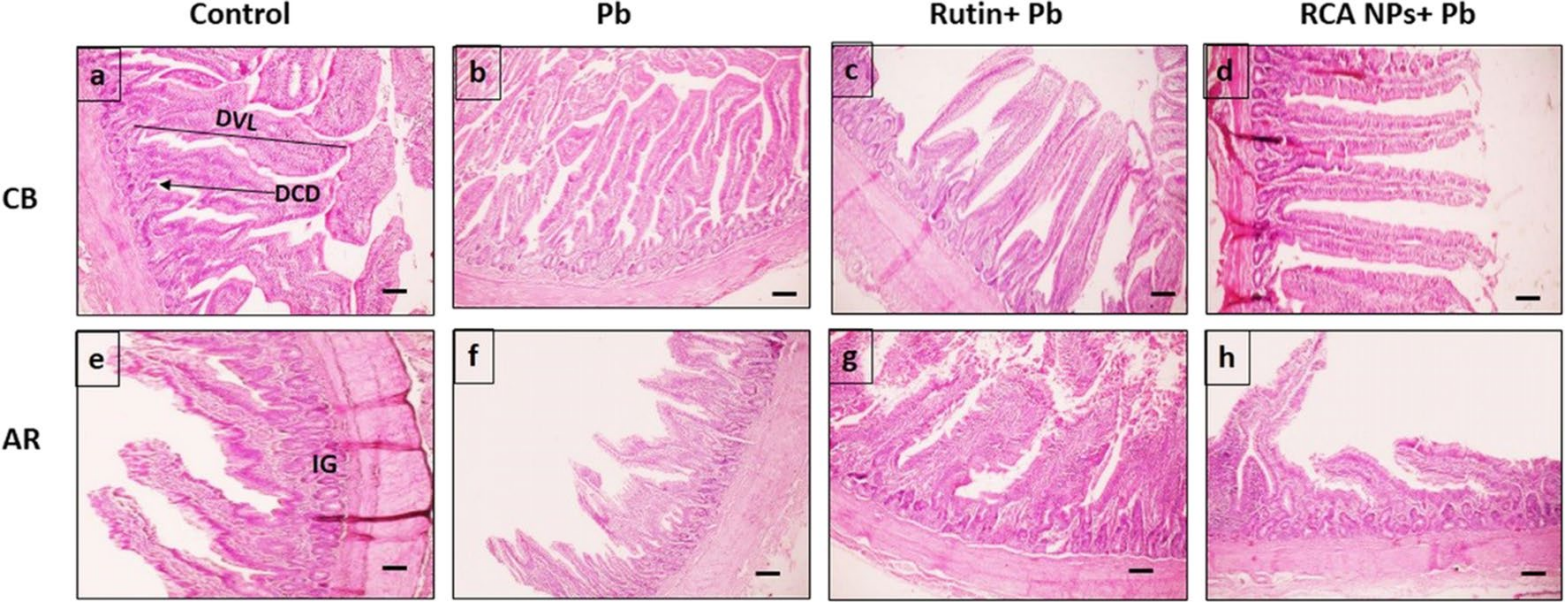
Photomicrograph of duodenum sections stained with HE of CB and AR showing Control groups (**a** and **e**), Pb groups (**b** and **f**), Rutin+ Pb groups (**c** and **g**), and RCA NPs+ Pb groups (**d** and **h**). DVL=duodenal villi length, DCD= duodenal crypt depth, IG= intestinal glands. Scale bars= 100μm

**Fig. 5:**
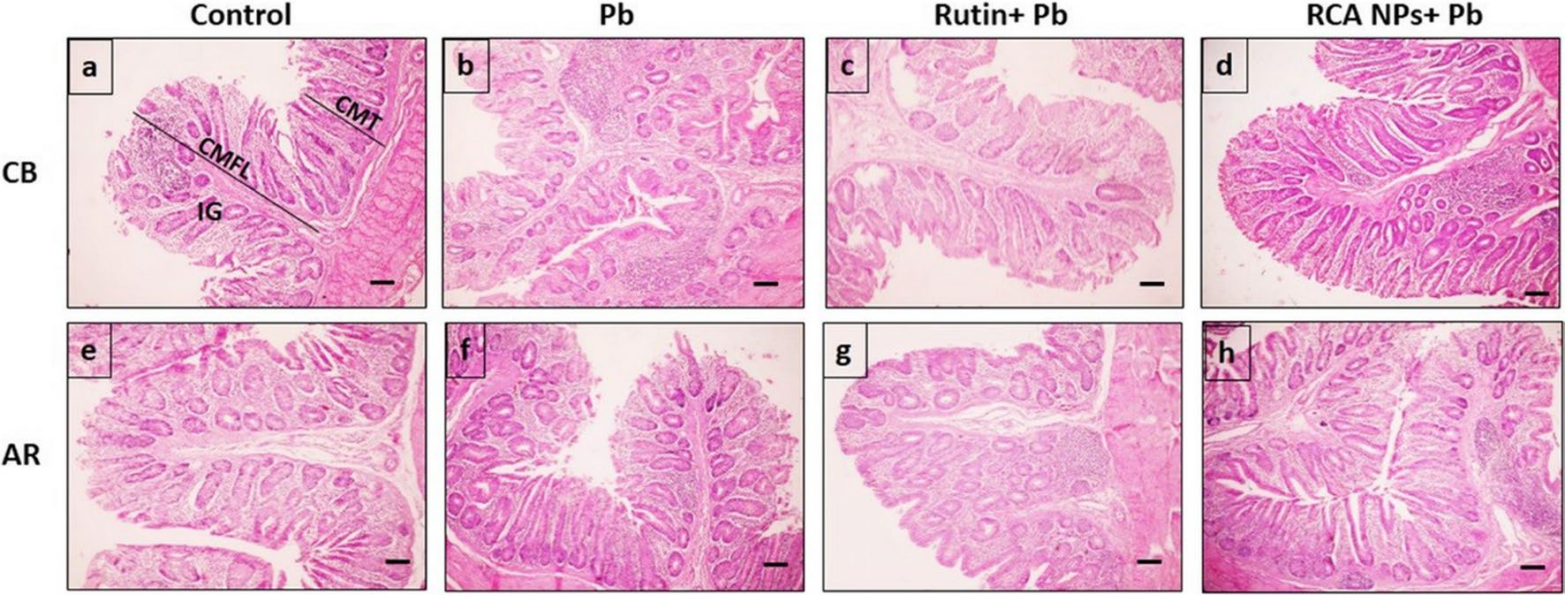
Photomicrograph of cecum sections stained with HE of CB and AR showing Control groups (**a** and **e**), Pb groups (**b** and **f**), Rutin+ Pb groups (**c** and **g**), and RCA NPs+ Pb groups (**d** and **h**). CMFL= cecal mucosal fold length, CMT= cecal mucosal thickness, IG= intestinal glands, Scale bars= 100μm

Gut morphology is a crucial parameter for bird health and growth [115] as birds with short intestinal villi or folds have troubles in absorbing essential nutrients [116]. In the present study, examination of intestinal tissue of control groups of CB breed (Fig. 4a) and AR breed (Fig. 4e) showed the duodenal mucosal layer with varying villi lengths (DVL) and crypt depths (DCD). Villi in Pb-treated groups appeared thin with sloughed parts (Fig. 4b and f), but rutin- and RCA NPs-treated groups had a very similar histological structure to the control group (Fig. 4c, d, g, and h). Based on histomorphometric analysis, Pb-treated groups showed the lowest value in DVL and DCD while RCA NPs-treated groups had the tallest villi, and deepest crypts (Fig. 7a and b). Furthermore, the cecal mucosal layers revealed that the mucosal folds were measured along with the tunica mucosa thickness since control groups of both breeds had tall folds with thick mucosa (Fig. 5a and e) while Pb-treated groups had short folds and thin mucosa (Fig. 5b and f). On the other hand, rutin- and RCA NPs-treated groups of both breeds had nearly similar structure to control groups (Fig. 5c, d, g, and h). The cecal mucosal fold length (CMFL) and tunica mucosa thickness (CMT) showed significant differences between groups (*P* < 0.01) whereas Pb-treated groups showed the shortest folds and the thinnest mucosal layer while RCA NPs-treated groups showed the thickest mucosal layer and the longest folds (Fig. 7c and d). Moreover, number of intestinal glands/fields in both duodenal and cecal tissues was counted in all treated groups; rutin- and RCA NPs-treated groups showed significant increase in number of intestinal glands compared Pb-treated groups since Pb-treated CB breed had the fewest glands (Fig. 7e). These findings revealed that Pb could induce necrosis and desquamation of intestinal villi with subsequent loss of nutrients and weight gain [117]. However, adding rutin to broilers’ diets increased DVL, DVL/DCD ratio, and villi area, resulting in better intestinal function and improved bird growth performance [118]. Moreover, [54] showed that chitosan supplementation at different levels could increase crypt depth and intestinal mucosa thickness, which ensured full-absorption of nutrients.

**Fig. 6:**
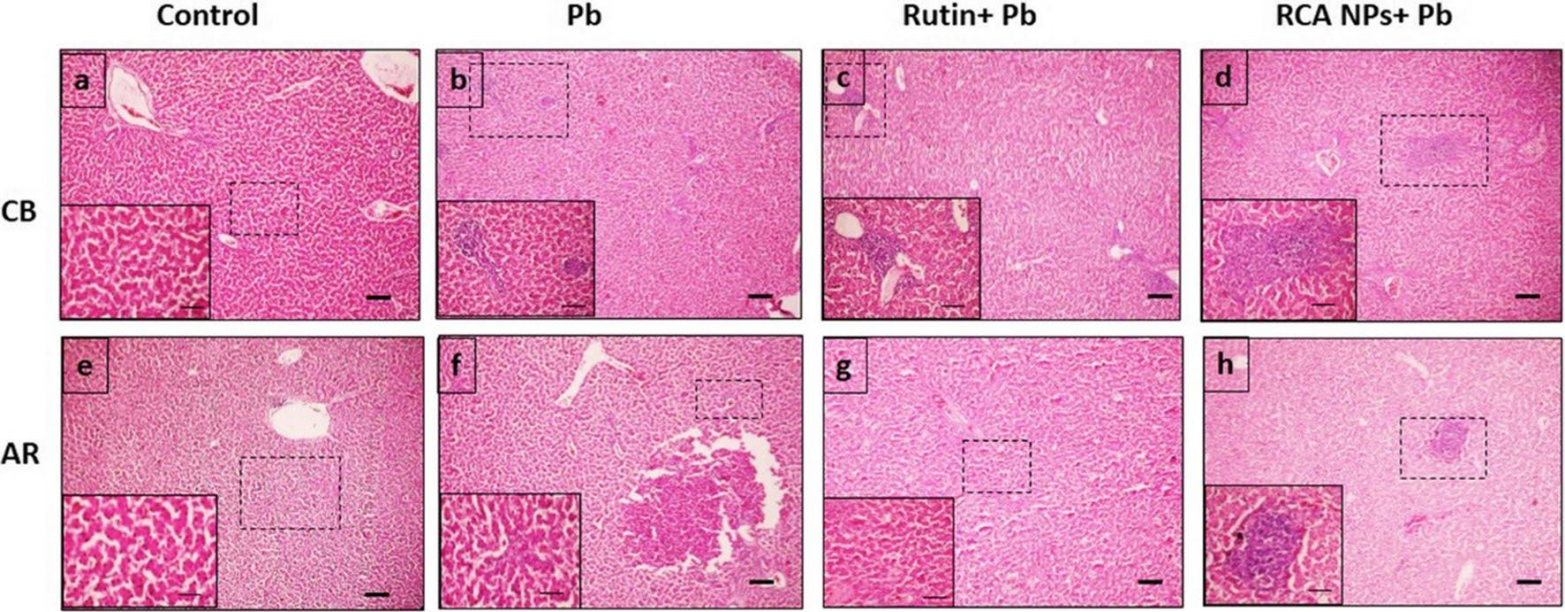
Photomicrograph of liver sections stained with HE of CB breed and AR breed showing Control groups (**a** and **e**), Pb groups (**b** and **f**), Rutin+ Pb groups (**c** and **g**), and RCA NPs+ Pb groups (**d** and **h**). Scale bars= 100μm and 50μm for magnified insets

**Fig. 7:**
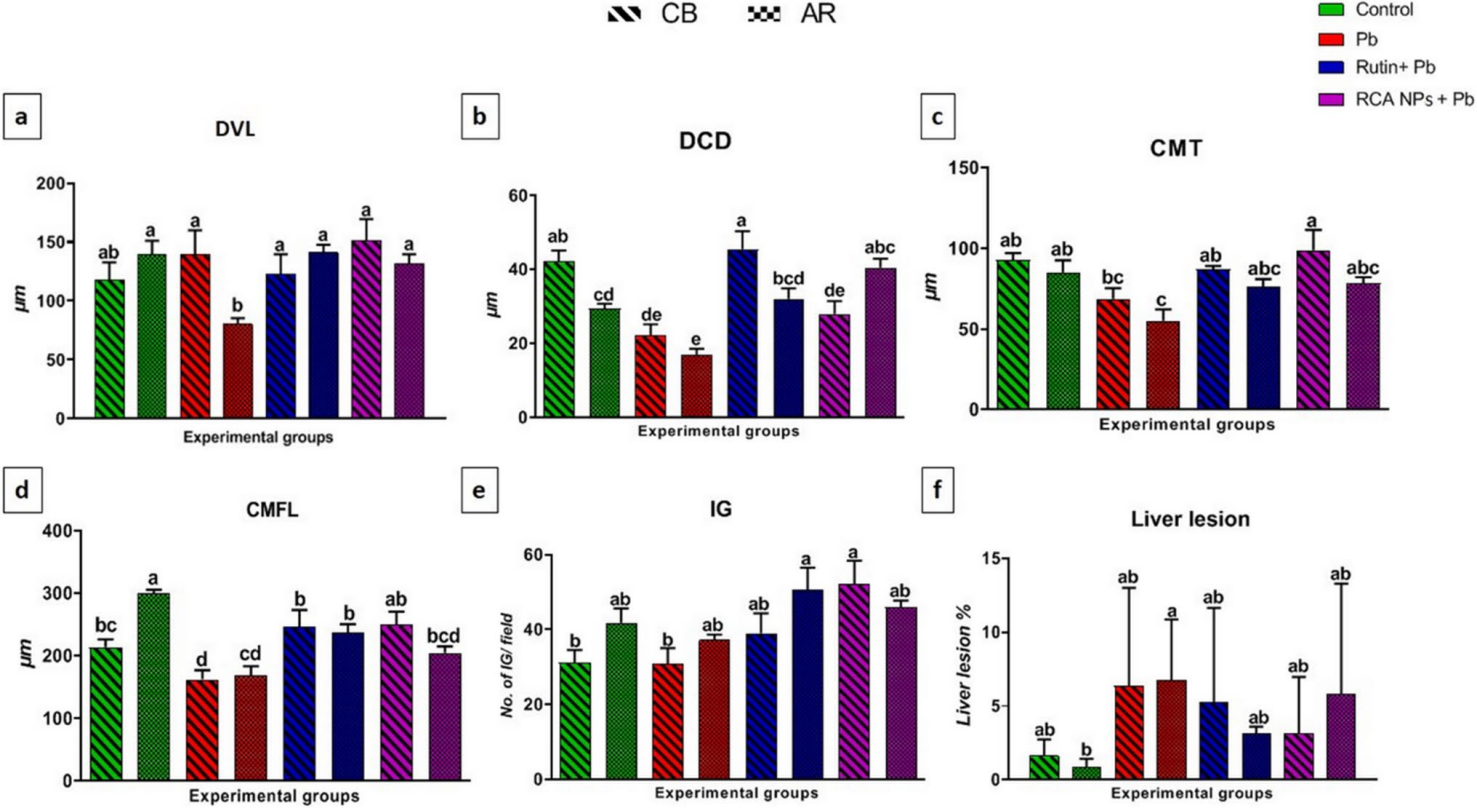
Charts showing different measurements, intestinal evaluation of tested groups using DVL (**a**), DCD (**b**), CMT (**c**), and CMFL (**d**), and IG number/field (**e**) in addition to liver lesion % (**f**). Data analyzed through two-way analysis of variance (ANOVA) and different letters indicate statistical significance at (*P* < 0.01)

Liver also plays a key role in metabolism and health of birds so any structural and functional abnormalities in the hepatic tissue could induce negative effects on growth performance [119]. The current study displayed that liver tissue of the control groups showed cords of normal hepatocytes separated by hepatic sinusoids. In contrast, Pb-treated groups showed hepatic degeneration and leukocytic infiltration that moderately resolved in rutin- and RCA NPs-treated groups (Fig. 6 and Fig. 7f). These findings coincided with previous study [120] which revealed that Pb at dose level 50mg/kg feed in CB broiler could interfere with the antioxidant defense mechanism and generating ROS, which mimic degenerative changes and inflammation in the hepatic tissue, while rutin has a good therapeutic effect on liver injury that may be associated with not only its potent antioxidative and anti-inflammatory effects but also through inhibiting lipogenesis and enhancing fatty acid metabolism [95, 121].

## Conclusion

Based on the findings of this study, it can be concluded that RCA NPs supplementation at dose level of 50 mg/kg feed could enhance profitability and growth performance within poultry farming operations exposed to environmental stressors. This improvement is mainly attributed to its positive effects on biochemical parameters, antioxidant enzyme activities, gene expression of growth-associated genes and morphological structure of intestinal and liver tissue. Furthermore, breed type could affect growth performance and resistance to various stressors with subsequently economic outcomes since a notable improvement was prominent in AR broiler breed with superior outcomes more than CB breed. Therefore, rutin supplementation especially in the form of RCA NPs is recommended to improve the overall productivity and profitability in poultry farms to encounter challenges associated with oxidative stress, such as Pb toxicity. Moreover, Further studies should be conducted to evaluate the efficiency of RCA NPs supplementation against other immunosuppressive stressors.

## Data Availability

Data are available upon request from corresponding author.
